# The global threat of Zika virus to pregnancy: epidemiology, clinical perspectives, mechanisms, and impact

**DOI:** 10.1186/s12916-016-0660-0

**Published:** 2016-08-03

**Authors:** Phillipe Boeuf, Heidi E. Drummer, Jack S. Richards, Michelle J. L. Scoullar, James G. Beeson

**Affiliations:** 1 Centre for Biomedical Research, Burnet Institute, Melbourne, Australia; 2 Department of Medicine, The University of Melbourne, Melbourne, Australia; 3 Department of Microbiology, Monash University, Clayton, Australia; 4 Department of Microbiology and Immunology, The University of Melbourne, Melbourne, Australia

**Keywords:** Biology, Pregnancy, Microcephaly, Placenta, Epidemiology, Economic cost, Pathogenesis, Public health

## Abstract

Zika virus (ZIKV) is a mosquito-borne flavivirus that has newly emerged as a significant global threat, especially to pregnancy. Recent major outbreaks in the Pacific and in Central and South America have been associated with an increased incidence of microcephaly and other abnormalities of the central nervous system in neonates. The causal link between ZIKV infection during pregnancy and microcephaly is now strongly supported. Over 2 billion people live in regions conducive to ZIKV transmission, with ~4 million infections in the Americas predicted for 2016. Given the scale of the current pandemic and the serious and long-term consequences of infection during pregnancy, the impact of ZIKV on health services and affected communities could be enormous. This further highlights the need for a rapid global public health and research response to ZIKV to limit and prevent its impact through the development of therapeutics, vaccines, and improved diagnostics. Here we review the epidemiology of ZIKV; the threat to pregnancy; the clinical consequences and broader impact of ZIKV infections; and the virus biology underpinning new interventions, diagnostics, and insights into the mechanisms of disease.

## Background

**Fig. 1:**
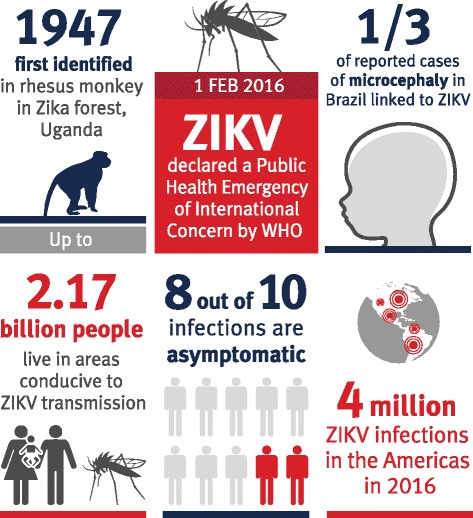
Zika virus infection has rapidly emerged as a significant global threat. See text for further details

Zika virus (ZIKV) infection was previously considered to be of modest public health concern, causing only mild fever, rash, and arthralgia in 20 % of patients, with 80 % of infections being asymptomatic [1]. Recently, ZIKV has caused large outbreaks in the Pacific (especially in the island of Yap in 2007 and in French Polynesia in 2012–14). The subsequent major outbreaks in Central and South America (especially Brazil) in 2015–16 led to the World Health Organization (WHO) declaring the situation as a Public Health Emergency of International Concern, placing it on the same priority list as the recent Ebola virus outbreak (Fig. 1). Increase in research efforts has led to ZIKV now being an accepted cause of the major increase in neurological abnormalities, including microcephaly and Guillain–Barré syndrome, reported in these regions.

## Zika virus epidemiology

ZIKV is a flavivirus that is primarily transmitted by daytime-active mosquitoes of the *Aedes* spp. Other routes of transmission have been reported, including sexual transmission, with low rates of transmission from oral and anal sex also described [2–7]. ZIKV was first identified in a rhesus macaque in the Zika forest in Uganda in 1947 [8]. The first evidence that ZIKV could infect humans came from serological surveys conducted in Uganda [9]. Evidence of sporadic human infections was then demonstrated across Africa and parts of South-East Asia, but the first major ZIKV outbreak described was in 2007 on the island of Yap in the Federated States of Micronesia. In that epidemic, it was estimated that >73 % of the total population was infected [1]. Since 2007, ZIKV has continued its eastward migration, detected in French Polynesia in 2012–14, the Easter Islands (Chile) in 2014, and Brazil in 2015, where between 500,000 and 1.5 million cases of Zika have occurred. ZIKV transmission has now been documented in 66 countries and territories; since 2015, 49 countries and territories previously ZIKV-negative have experienced their first reported ZIKV outbreak [10].

## Clinical manifestations

**Fig. 2:**
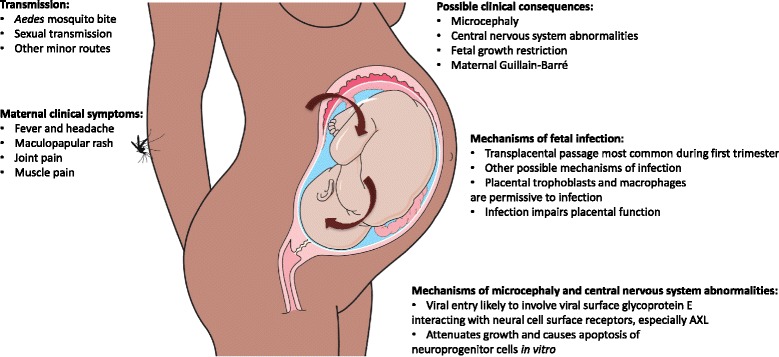
Zika virus disease pathogenesis. The figure summarizes key points regarding ZIKV transmission, clinical features and complications, and mechanisms of fetal infection and microcephaly and central nervous system abnormalities

Microcephaly has emerged as an accepted consequence of ZIKV infection during pregnancy. It describes a fetus or infant with a head circumference (HC) smaller than expected for gestation or age, and is usually categorized as primary microcephaly (mainly from genetic causes) or secondary microcephaly (nongenetic causes such as infection or disruption of brain vasculature) [11, 12]. The criteria used to define microcephaly vary. The more stringent definition is a HC of three standard deviations (SD) below the mean. This definition includes all those with clinically significant microcephaly that is highly likely to be associated with severe developmental delay, intellectual impairment, and other severe complications. A less stringent definition of a HC of two SD below the mean is currently used in Brazil for the postnatal diagnosis of microcephaly. This is practical given that around 33 % of infants with a HC between two and three SD below the mean have moderate to severe intellectual impairment [13]. In the context of ZIKV infection, the real need for consensus around the definition of microcephaly is most important for its in utero diagnosis. This is a technically difficult task that has been aided by the recently released guidelines from the Society for Maternal Fetal Medicine [14]. Other structural cerebral abnormalities are also associated with congenital ZIKV syndrome, including brainstem and cerebellar hypoplasia, delayed myelination, severe ventriculomegaly, gross calcification of the brain parenchyma, and some cases of lissencephaly (absence of normal cerebral folds) [15, 16]. Unlike other viruses in pregnancy that are associated with a more generalized congenital syndrome affecting a number of different organs, ZIKV appears to predominantly affect neural tissues. Two recent reports of neuroimaging in a total of 46 infants with likely ZIKV-associated microcephaly demonstrated severe brain damage in almost all cases [15–17]. Guillain–Barré is an additional serious complication that may follow ZIKV infection. Guillain–Barré syndrome is an immune-mediated disease affecting the peripheral nervous system that can lead to a severe peripheral neuropathy causing muscle paralysis, and can result in death in some cases [18]. Guillain–Barré syndrome has a range of potential causes, including other flaviviruses [19]; the association between ZIKV infection and Guillain–Barré syndrome in French Polynesia has now been clearly established [20]. However, this review will focus on ZIKV infection during pregnancy and the development of fetal microcephaly and other complications (Fig. 2).

## Level of threat to pregnant women

The rapidly expanding distribution of ZIKV and its potentially high penetrance in newly affected populations pose very significant threats to pregnant women and their fetuses in many regions. The first trimester of pregnancy is a crucial period for brain development and ZIKV infection early in pregnancy is likely to be more strongly associated with microcephaly than infections later in pregnancy, as demonstrated in French Polynesia [21]. Consequently, the impact of the expansion of ZIKV to previously unexposed populations may not be perceived for a number of months, as seen in the delayed observations of microcephaly and fetal malformations in French Polynesia [21] and Brazil [22]. As of 25 June 2016, in Brazil, there were 8165 reported cases of microcephaly suspected to be associated with ZIKV infection, of which 5104 have been investigated and 1638 (32 %) were confirmed to be linked to ZIKV [23]. Modeling data from French Polynesia revealed that the risk of microcephaly from infection in the first trimester was 1 % [21]. This estimation relied on passive surveillance of only ~30 % of general practice clinics and is likely to be an underestimation of the actual risk given that some mothers could have had a miscarriage or stillbirth or may not have presented for clinical care. Such data from the Brazil outbreak are not yet available. This relatively low risk has to be balanced with the large population exposure and high incidence of ZIKV in some regions (e.g., ~70 % in French Polynesia [21] and Yap [1]). The reasons why microcephaly and other congenital complications associated with ZIKV have become prominent in recent outbreaks is currently unclear. It may be explained by specific mutations in ZIKV strains causing recent epidemics, and/or host or other cofactors. The enormous size of recent and ongoing epidemics has also aided the detection of complications.

## Strength of the evidence for a link between Zika virus and microcephaly

Evidence for a causal link between ZIKV and microcephaly is now very strong and widely accepted [24] (Table 1). Despite the lack of data from adequately powered prospective longitudinal studies, the link between ZIKV and microcephaly is supported by applying Shepard’s Criteria for Proof of Teratogenicity in Humans [24] and the Bradford–Hill criteria for causality [25], especially for the aspects of temporality, biological plausibility, and analogy. For example, temporality of the association is supported by individual case reports as well as the ~6-month delay between ZIKV outbreaks and the increase in the incidence of microcephaly in French Polynesia [21] and Brazil [22], suggesting a causal link between microcephaly and ZIKV infection in the first/early second trimester. Moreover, modeling of French Polynesia cases demonstrated that “the best-fitting models of period-of-risk all included the first trimester of pregnancy, with that including only the first trimester having the best fit” [21], even if central nervous system abnormalities have been reported for fetuses infected as late as 27 weeks of gestation [26]. Plausibility is supported by the detailed study of an aborted microcephaly case from Ukraine for which other infectious causes of microcephaly were ruled out [27]. This case had evidence of ZIKV infection in the fetal brain, suggesting ZIKV can cross the fetal blood–brain barrier. This is supported by reports of vertical transmission of ZIKV in a mouse model, leading to impaired fetal brain development [28]. Furthermore, ZIKV can infect human neural progenitor cells and attenuate their growth [29]. These findings have been replicated in a mouse model in which ZIKV targets primarily neural progenitor cells, causing their cell-cycle arrest, apoptosis, and inhibition of differentiation, resulting in cortical thinning and microcephaly [30]. This and other animal models have recently provided evidence that ZIKV infection can lead to microcephaly (reviewed in [31]), indicating that a causal link between early pregnancy ZIKV infection and microcephaly is plausible in humans (see below for possible mechanisms). Mouse models point to the importance of a type I IFN response in the susceptibility to ZIKV infection and in the development of clinical symptoms (including microcephaly) [32, 33]. The relevance of these findings in humans is unclear. There is also an analogy between ZIKV and other viruses (including flaviviruses) for which a link with microcephaly has been demonstrated; for example, rubella can cause microcephaly with cerebral calcifications [34], as described with ZIKV infection [27] and cytomegalovirus [35].

**Table 1:** Evidence for a causal link between ZIKV and microcephaly

*Epidemiological and clinical findings*	
	Increase in microcephaly cases coincides with increase in ZIKV transmission (with a 6-month delay)
	Data modeling shows that the main period at risk is the first trimester of pregnancy
	Of the microcephaly cases investigated in Brazil, 32 % were linked to ZIKV
	Case study: Miscarriage of a baby with microcephaly was positive for ZIKV (including in its brain), but negative for other known infectious causes of microcephaly
*Laboratory studies*	
	ZIKV can infect human neural progenitor cells and attenuate their growth in vitro
	Primary human placental macrophages and trophoblasts are permissive to ZIKV infection in vitro
*Animal models*	
	Mouse model of ZIKV display signs of microcephaly
*Analogy to related viruses*	
	Rubella virus, another flavivirus, causes microcephaly when infection occurs during pregnancy

Note: See text for further details and discussion of the evidence

These and other lines of evidence collectively support that ZIKV can cause microcephaly and led the Centers for Disease Control and Prevention (CDC) to conclude that “sufficient evidence has accumulated to infer a causal relationship between prenatal Zika virus infection and microcephaly and other severe brain anomalies” [24].

## Consequences of Zika virus-associated microcephaly on public health and health economics

The WHO predicts up to 4 million ZIKV infections in 2016 in the Americas alone [36] and initial modeling suggests that up to 2.17 billion people live in areas conducive to ZIKV transmission [37]. Given the potential enormous scale of the issue, greater attention needs to be given to the public health consequences and likely economic impacts of an increased number of children with microcephaly and other neurological and ocular abnormalities [38, 39]. These abnormalities, as well as microcephaly, are strongly associated with intellectual impairment, seizures, visual and hearing impairment, feeding difficulties, and significant developmental delay, signifying a poor prognosis for affected children with many unable to talk or walk [40–42]. As mentioned above, ZIKV infections in adults have also been linked with Guillain–Barré syndrome, a potentially debilitating and serious complication.

ZIKV could potentially impact on the economies of affected countries, such as through reduced tourism and impact on trade [43]. However, there has been little focus on the economic impact to the health system and implications regarding cost of treatment, and the loss of productivity of affected children and their carers. Disability is associated with lower educational attainment, higher unemployment, and additional financial costs for families [44]. These financial costs are at times over 40 % of household income, representing a potentially catastrophic health expense that may drive some households into poverty and perpetuate the cycle of entrenched poverty and poor health [44, 45]. Providing appropriate healthcare and support services for these children will further stretch already overburdened health systems. Quantifying this burden on a society, health system, and economy is difficult due to the paucity of available data, but it is likely to be substantial. Evidence emerging from Brazil describes families struggling to access appropriate specialist services and treatment, a situation further exacerbated by Brazil’s current economic downturn [46, 47]. Dengue virus, a related flavivirus also transmitted by *Aedes* spp., caused around 58 million symptomatic infections in 2013, and is thought to have cost US$8.9 billion to the global economy in 2013 [48]. The epidemiological and clinical differences between Dengue virus and ZIKV limit the generalizability of this cost estimate; however, it does highlight the potential scale of economies that could be at stake. For example, since January 2012, the Pacific region has experienced a major surge in mosquito-borne diseases with concurrent epidemics of dengue, chikungunya, and ZIKV infections affecting all 22 Pacific island countries and territories [49]. There is also a pressing need to assess the implications of ZIKV on global maternal and child health, and to ensure an equitable and accessible provision of quality, comprehensive care for affected families.

## Zika virus biology and vaccines

A strong knowledge of ZIKV biology is important for the development of vaccines, therapeutics, and better diagnostics. Recently, cryo-electron microscopy revealed the architecture of ZIKV strain H/PF/2013 isolated in Micronesia in the 2013–14 outbreak, confirming its structural analogy with other flavivirus members [50]. Similar to other flaviviruses, ZIKV is an icosahedral enveloped virus with an ~11 kb positive-sense RNA genome encoding a single polyprotein of 3417–3423 amino acids. The polyprotein encodes three structural proteins [capsid (C), precursor membrane (prM), and envelope protein (E)] and seven nonstructural proteins (NS1, NS2A, NS2B, NS3, NS4A, NS4B, and NS5 proteins), which are derived by cleavage of the large polyprotein by proteases. By analogy to other flaviviruses, viral entry of ZIKV into host cells is assumed to involve the specific binding of viral surface glycoprotein E to cellular receptors, followed by endocytosis, viral fusion, and delivery of the nucleocapsid into the cytoplasm. Viral replication occurs in the cytoplasm and viral assembly occurs in the endoplasmic reticulum [51]. The ectodomain of the ZIKV glycoprotein E comprises a typical three-domain organization (domain I, II, and III). The fusion loop resides at the tip of domain II and is important for viral fusion with the host-cell membrane during viral entry. Domain III is connected via the stem region to the transmembrane domain (TMD) that anchors the glycoprotein E to the viral membrane [50, 52, 53]. The region surrounding the Asn154 glycan of the glycoprotein E shows the biggest structural deviation from the dengue virus envelope glycoprotein as a result of an insertion of two N-terminal and four C-terminal residues flanking Asn154. In the related West Nile virus, the Asn154 glycan is associated with neurovirulence in isolates obtained post-1999; mouse-adapted strains with poor neurovirulence lack a glycan at this position [54]. Further study on the role of the Asn154 glycan in ZIKV are required to examine whether it plays a role in ZIKV neurovirulence. Understanding the nature and function of these proteins and glycans is important for developing novel anti-ZIKV drugs and vaccines.

At this stage, a number of vaccine approaches are being pursued, including DNA-based and recombinant protein subunit vaccines. Live attenuated vaccines are also promising, being modeled off work for yellow fever and dengue viruses. Given recent findings in animal models, a vaccine for ZIKV appears to be a feasible proposition. A study conducted in Balb/c mice showed that a single vaccination of DNA encoding the full-length prM-E region of the BeH815744 strain from Brazil afforded complete protection 4 or 8 weeks later from a challenge dose of 10^2^ plaque-forming units (pfu) of either a ZIKV isolate from Brazil (ZKV2015) or an isolate from Puerto Rico (PRVABC59), two strains from the Asian lineage that differ by five amino acids [55]. Truncation of the prM-E region to remove either the TMD of glycoprotein E or the TMD and stem region of glycoprotein E, or immunization with full-length glycoprotein E alone, TMD-truncated glycoprotein E, or TMD and stem-truncated glycoprotein E were not protective; however, viral loads were lower compared to those in sham-vaccinated animals. Analysis of the correlates of protection revealed that glycoprotein E-specific antibody titers correlated with protection and inversely correlated with viral load in challenged animals. The protective efficacy of this vaccine was mediated by IgG, as demonstrated by the fact that passive transfer of IgG into naïve animals was protective, and depletion of CD4^+^ T cells, CD8^+^ T cells, or CD4^+^ and CD8^+^ T cells did not alter the level of protection afforded by the vaccine.

These observations were extended to a conventional vaccine platform using an inactivated purified ZIKV vaccine (Puerto Rico PRVABC59 strain). Immunization of Balb/c mice with the vaccine (formulated with alum as the adjuvant) generated neutralizing antibodies and all animals were protected when challenged with the Brazilian ZKV2015 strain [55]. It is important to note that the Brazilian ZKV2015 strain used in these challenge studies has been demonstrated to cause fetal microcephaly and intrauterine growth restriction in wild-type SJL mice [56]. In addition, while the Balb/c mice used by Larocca et al. [55] did not have a fatal outcome from infection, viremia was detected and its duration was similar to that observed in human infections of ZIKV. How these findings will translate to humans and the prevention of microcephaly is unclear, but these findings provide a proof of concept that it will be possible to develop a ZIKV vaccine for clinical evaluation in humans using a nonreplicating vaccine.

## Diagnosis of Zika virus infection

The current tools available to accurately diagnose ZIKV infections are limited, and there is a need for highly specific point-of-care tests for ZIKV detection and improved serological tools for clinical diagnosis and population-based surveillance [57, 58]. Available diagnostics have mainly been used to test symptomatic individuals and it is unclear how they perform in asymptomatic individuals, who comprise ~80 % of infected individuals and who may be important reservoirs for virus transmission [1]. These diagnostic uncertainties are especially important for pregnant women who may infect their fetus, with severe consequences for fetal development, despite having asymptomatic infections themselves. Therefore, and in accordance with recent CDC guidelines for testing pregnant women with possible ZIKV exposure, there is a strong justification for testing asymptomatic at-risk pregnant women as long as laboratory capacity is adequate [59]. These at-risk women include those who have traveled to areas with active ZIKV transmission and those who have had sex without a condom with a male partner with possible ZIKV exposure, especially if they or their partner develop symptoms or signs of ZIKV disease.

Anti-ZIKV antibodies are highly cross-reactive as a result of antigenic relatedness between ZIKV and other flaviviruses such as dengue, Japanese encephalitis, West Nile virus, Kunjin virus, yellow fever, Murray Valley encephalitis, and others that often co-circulate with ZIKV [60]. Cross-reactivity with antibodies induced from vaccination with yellow fever and Japanese encephalitis vaccines can also occur [61]. These limitations in current serological assays remain a major obstacle for individual diagnostics and screening, as well as population-based serosurveillance. The detection of circulating antigen(s) is currently not available for ZIKV, but this approach is likely to be valuable because the period of antigenemia extends beyond the period of viremia. In dengue, detection of NS1 antigen has been used for direct virus detection, and IgM-specific reactivity adds specificity. Various technology platforms are exploring ways of multiplexing serological responses to a range of flaviviruses and other co-circulating arboviruses [62, 63].

As with other arboviruses, viral culture itself is possible, but is impractical for routine diagnostic use. Plaque reduction neutralization assays (PRNT) may be used as confirmatory assays if there is no prior evidence of previous flavivirus infection or vaccination with a flavivirus vaccine as the potential for cross-reactivity of antibodies acquired from exposure to other flaviviruses can be problematic. Nucleic acid amplification methods like reverse transcription (RT) PCR remain the main diagnostic approach in acutely symptomatic individuals owing to their high sensitivity and specificity. The viremia period extends from a brief pre-patent period and commonly continues for 3–5 days after the onset of symptoms [64–66], but may extend for up to 11 days [60, 67]. RT-PCR can also be used to detect ZIKV in amniotic fluid [68], breast milk [69, 70], semen [71], and saliva [72], and may be used to screen blood products. Given that ZIKV can be found in urine for up to 7 days longer than in serum, urine may be a more useful sample when there are delays in presentation [73, 74]. With proven or suspected microcephaly, testing for other etiological factors is important, including rubella, cytomegalovirus (CMV), toxoplasmosis, herpes simplex virus, varicella zoster virus, HIV, and chikungunya virus, as well as excluding other noninfectious causes. PCR assays are often used with consensus primers targeting multiple flaviviruses, followed by sequencing or ZIKV-specific PCR. Generic flavivirus PCRs may suffer from lower sensitivity compared to species-specific PCR, but it provides a practical screening tool, especially when a range of other infections need to be considered.

## Potential mechanisms linking Zika virus and microcephaly

Microcephaly developing in utero (hereafter microcephaly) is principally due to impaired neurogenesis, which includes a reduced number of neural progenitor stem cells and/or impaired neuronal division and differentiation [75]. Several noninfectious causes or conditions associated with microcephaly have been described, including genetic predisposition [76] and prenatal alcohol exposure [77]. Supporting ZIKV infection as a cause of microcephaly, ZIKV has been detected in the brain tissue of microcephalic fetuses [27, 78] and in vitro evidence demonstrates that ZIKV can infect neural progenitor cells and attenuate their growth [29]. Infection of human cortical neural progenitor cells can cause cell cycle dysregulation and caspase-3-mediated apoptosis [29]. Permissiveness of cells for ZIKV entry appears to be supported by several surface receptors, including DC-SIGN, AXL, Tyro3, and, to a lesser extent, TIM-1, with a major role for AXL [79]. In fetal brain tissue, AXL is highly expressed in cells of the developing cerebral cortex, including radial glial cells, astrocytes, endothelial cells, and microglia, but the expression of Tyro3 and DC-SIGN is low or absent, respectively [80]. Expression of AXL in the outer margin of the neural retina and in cells of the ciliary marginal zone adjacent to neural retina provides a possible explanation for the development of blindness in babies born to ZIKV-infected mothers [80, 81]. After entry of the virus, replication of viral RNA induces a strong antiviral response with upregulation of TLR3 mRNA as well as RIG-I and MDA-5 mRNA. Silencing of TLR3 causes strong upregulation of viral replication but does not alter the type I interferon (IFN) response. Treating infected cells with IFN-α, IFN-β, or IFN-γ caused a dose-dependent inhibition of viral replication [79], which may suggest potential therapeutic approaches for future development.

ZIKV could reach the fetal brain by transplacental passage and/or diffusion into the amniotic and yolk sacs during embryogenesis [82]. A transplacental passage is plausible, even if term placental cells appear to be protected against ZIKV infection by a constitutive IFN-**λ** response [83]. It is not known whether these protective mechanisms are in place and efficient in early pregnancy. A recent study showed that primary human placental macrophages and trophoblasts were permissive to productive ZIKV infection [84]. This in vitro evidence is supported by ex vivo findings of ZIKV detection in chorionic villi. This demonstrates that ZIKV can infect the placenta, most likely from maternal blood. Other routes of fetal ZIKV infection early in pregnancy could include leakage through the trophoblastic plugs or diffusion of ZIKV into the amniotic and yolk sacs as they form. ZIKV has been identified in semen, which could give the virus access to the early embryo given the strong evidence for sexual transmission of ZIKV [85]. However, this is unlikely to be the main route of embryonic infection.

Placental ZIKV infection could impair placental functions, contributing to the fetal growth restriction and placental insufficiency sometimes described for ZIKV infections associated with microcephaly [21, 22, 27]. In particular, aberrant placental autophagy could contribute to an impairment of placental functions. In placental cells, autophagy usually limits viral replication [86]. However, in skin fibroblasts, ZIKV appeared to stimulate autophagy, which is associated with higher ZIKV loads [79].

## Conclusions

ZIKV has recently emerged as a major global threat to pregnancies, and there is now strong evidence linking ZIKV infection with microcephaly and other significant congenital abnormalities. Given the serious congenital complications that can arise from ZIKV infection and the substantial long-term consequences of these, a strong and rapid global public health and research response to the virus is essential to limit and prevent the major health, social, and economic impact of the virus, and to advance the development of therapeutics, vaccines, and improved diagnostics. This will require substantial ongoing funding commitments. Moreover, the current Zika crisis is a salient reminder of the ongoing threat to human health posed by infectious pathogens.
